# Regulation of Autophagy, Apoptosis, and Cell Death in Cardiomyocytes and Potential Therapeutic Targets

**DOI:** 10.31083/RCM52423

**Published:** 2026-10-10

**Authors:** Xing Feng, Ye Wang, Tangna Sun, Tongku Liu

**Affiliations:** ^1^Department of Cardiology, Jilin People’s Hospital, 132001 Jilin, Jilin, China; ^2^The Center of Cardiology, Affiliated Hospital, Beihua University, 132011 Jilin, Jilin, China

**Keywords:** cardiomyocytes, autophagy, apoptosis, necrosis, regulatory mechanisms, therapeutic targets

## Abstract

Disruption of cardiomyocyte homeostasis is a fundamental pathological basis for the onset and progression of cardiovascular diseases (CVDs). Given the extremely limited regenerative capacity of cardiomyocytes, programmed cell death (PCD) has emerged as a critical determinant of disease pathogenesis and clinical outcomes. PCD comprises multiple forms of cell death, including necroptosis, pyroptosis, ferroptosis, cuproptosis, disulfidptosis, necrosis induced by sodium overload (NECSO), lysosomal cell death, oxeiptosis, PANoptosis, entotic cell death, netotic cell death, alkaliptosis, and poly(ADP-ribose) polymerase-1 (PARP-1)-dependent cell death (parthanatos). Complex molecular interactions and signaling crosstalk among these cell death pathways can lead to the dysregulation of these pathways and, in turn, induce various CVDs. Currently, the mechanisms linking cardiomyocyte PCD to CVDs remain incompletely understood. Therefore, this article systematically reviews advances in research over the past decade on autophagy, apoptosis, necrosis, and other forms of PCD in cardiomyocytes. Moreover, this review summarizes the molecular mechanisms and pathway interaction networks underlying these cell death modalities, examines key regulatory targets and the potential to develop and translate targeted inhibitors into clinical practice, and outlines strategies for targeted intervention in cardiomyocyte PCD to prevent and treat CVDs. Collectively, this review aims to provide a theoretical foundation for further research into precision therapies for heart diseases.

## 1. Introduction

Cardiovascular disease (CVD) is a leading cause of morbidity and mortality worldwide. Myocardial cell death plays a critical role in the complex pathophysiological mechanisms underlying the onset and progression of CVD.

Cell death is broadly classified into accidental cell death (ACD), traditionally regarded as non-programmed and unregulated, and regulated cell death (RCD) [1]. However, emerging evidence indicates that ACD is also governed by finely controlled processes [2,3], challenging the notion that ACD constitutes a distinct and purely accidental category. RCD refers to cell death that can be modulated, induced, accelerated, inhibited, or blocked by specific regulatory mechanisms. Key forms of RCD implicated in cardiomyocyte death include autophagy and autosis, a form of programmed cell death (PCD) distinct from apoptosis [4], necroptosis, pyroptosis [5], ferroptosis, cuproptosis [6], disulfidptosis [7], necrosis by sodium overload (NECSO) [8], lysosomal cell death [9], oxeiptosis [10], PANoptosis [11], entotic cell death, netotic cell death [12], alkaliptosis [13], and poly(ADP-ribose) polymerase-1 (PARP-1)-dependent cell death (parthanatos) (Fig. 1) [14]. In addition, although autophagy primarily serves as a homeostatic mechanism, this process can also contribute to cell death under certain conditions.

**Fig. 1.**
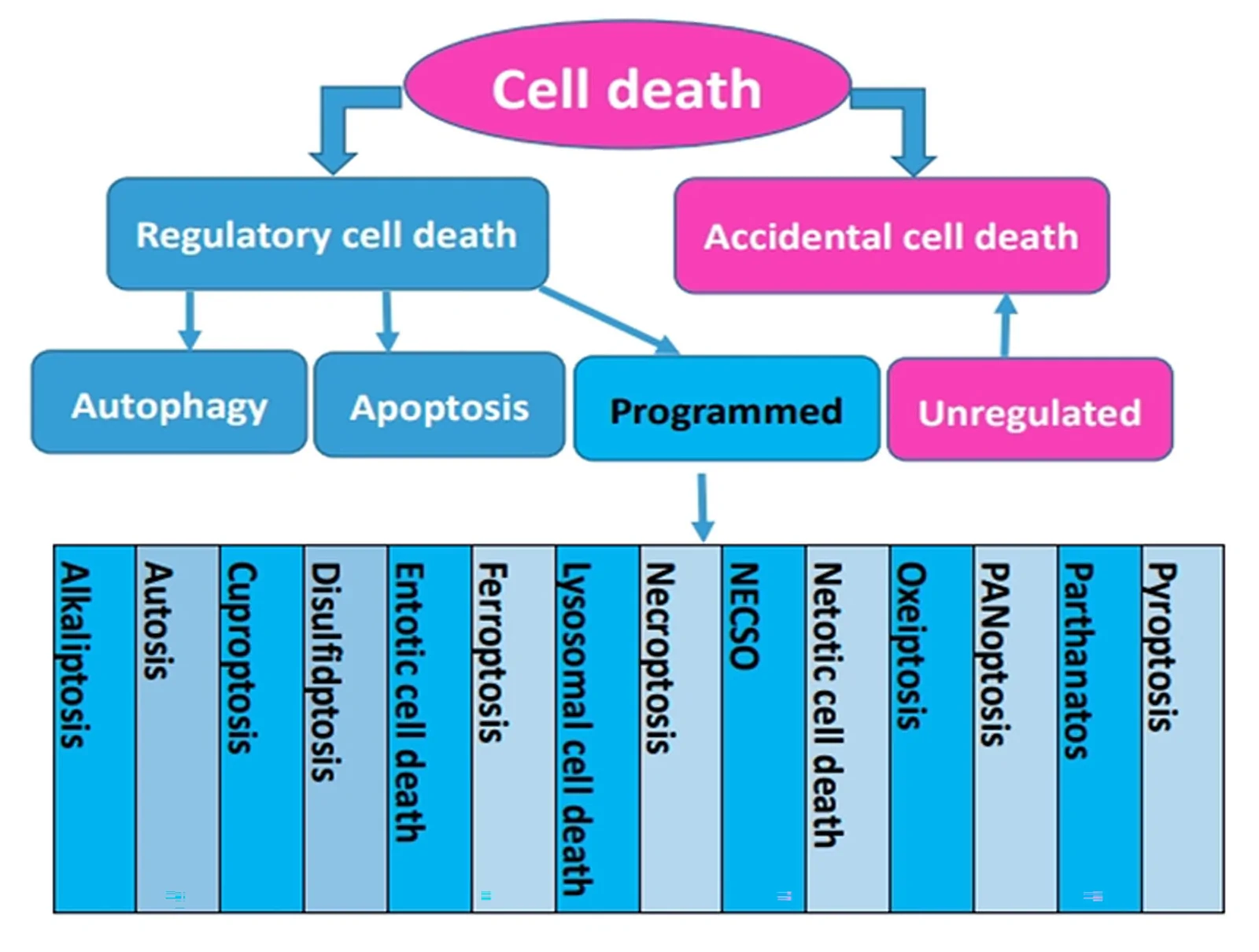
**Types of cell death**.

These RCD signaling pathways do not operate in isolation; rather, these pathways form a complex regulatory network that collectively influences cell fate and organ function. In the context of CVD, cell death modalities encompass not only RCD but also conventional forms of cardiomyocyte injury, such as inflammatory and necrotic cell death [15]. Notably, disulfidptosis, NECSO, and cuproptosis are novel RCD subtypes identified within the past five years. Thus, elucidating the mechanisms underlying these cell death modalities is critical for understanding the pathophysiological processes that drive the onset and progression of CVD and for identifying specific intervention targets for innovative cardioprotective and therapeutic strategies [16]. Nevertheless, most existing studies and reviews focus solely on individual RCD types and lack a comprehensive, systematic discussion of the crosstalk among various RCD patterns and the associated signaling pathways. This fragmented research landscape prevents clinicians from fully recognizing the roles of RCD in the initiation and progression of CVDs, and hinders investigations aimed at targeting key nodes within RCD signaling pathways. Specifically, review articles that systematically summarize multiple forms of RCD, especially newly identified subtypes reported in recent years, remain scarce. The lack of systematic literature that clearly elucidates the various cell death modalities and their regulatory signaling pathways severely constrains the understanding of clinicians regarding these mechanisms. Therefore, there is an urgent need for concise, clear, and accurate literature that describes cell death mechanisms and signaling pathways, enabling clinicians to quickly understand and stay abreast of the latest advances [4].

This article systematically reviews research advances over the past decade in cardiomyocyte death modalities, including apoptosis, necroptosis, pyroptosis, ferroptosis, and the interplay among these pathways, with an emphasis on the associated key molecular mechanisms and translational potential as therapeutic targets in CVD. Moreover, this review aims to serve as a reference for further in-depth investigations into the modalities of cardiomyocyte death and to support the development of targeted, precision therapies for CVD.

## 2. Autophagy in Cardiomyocytes: From a “Scavenger” to a Precisely Regulated System

### 2.1 New Concepts of Autophagy

Autophagy is a cellular process that mediates the removal of damaged organelles. Considerable research has focused on harnessing autophagy enhancement as a therapeutic strategy for various diseases [17,18]. During autophagy initiation, numerous vesicular structures known as autophagosomes form in the cytoplasm. These structures are delineated by a double membrane and sequester cytoplasmic components destined for degradation. Subsequently, autophagosomes fuse with lysosomes to form autolysosomes, in which the enclosed materials are degraded by lysosomal hydrolases into amino acids, nucleotides, and other basic metabolites. These degradation products enter the tricarboxylic acid (TCA) cycle to generate small molecules and adenosine triphosphate (ATP), which are then recycled by the cell [19,20]. Consequently, autophagy has long been considered a self-protective mechanism that promotes cell survival.

However, emerging evidence indicates that autophagy can also induce cell death under certain conditions. The autophagic process is tightly regulated by a range of genes, including autophagy-related genes (ATGs) and genes encoding protein kinases and phosphatases [21,22]. Within the cardiovascular system, basal autophagy is essential for maintaining cardiomyocyte homeostasis, whereas excessive activation or impaired autophagic flux contributes to pathological remodeling [23,24]. Recent studies have revealed that autophagy not only functions as a “scavenger” that adapts to energy stress and clears damaged organelles, but also serves as a key regulator involved in the pathogenesis of various heart diseases [25,26].

Autophagy is categorized into three distinct forms: macroautophagy, microautophagy, and chaperone-mediated autophagy. Macroautophagy is characterized by the formation of double-membrane autophagosomes that engulf cytoplasmic constituents and subsequently fuse with lysosomes to facilitate degradation, thereby enabling material recycling and organelle turnover [27,28]. Basal autophagy represents the physiological process by which cardiomyocytes perform routine quality control, removing misfolded proteins and dysfunctional mitochondria to preserve cardiac structure and function. Mice with cardiomyocyte-specific knockout of the essential autophagy genes *Atg5* or *Atg7* exhibit blocked autophagic flux, accumulation of abnormal proteins, mitochondrial dysfunction, and eventually develop progressive myocardial hypertrophy, contractile dysfunction, and heart failure in adulthood [29,30].

Cardiomyocyte death, whether through autophagy, apoptosis, or necrotic injury, impairs contractile and diastolic function, ultimately contributing to the development of heart failure [31,32]. Thus, developing targeted therapeutic strategies that modulate the regulatory networks governing excessive autophagy, apoptosis, and necroptosis holds promise for precision medicine [33,34] and remains a key focus of ongoing research.

### 2.2 Stress-Induced Autophagy: Adaptive Response and Dysregulation

Under mild stress conditions, such as moderate starvation or exercise, autophagy is upregulated to clear damaged cellular components and dysfunctional proteins, thereby maintaining cellular function and protecting the heart. However, under persistent or severe stress, autophagy regulation may become dysregulated [35,36]. In response to nutritional or energetic stress, the AMP-activated protein kinase/ubiquitin-like kinase 1 (AMPK/ULK1) pathway and the sirtuin family sense energy deficits and initiate autophagy. In conditions of myocardial energy metabolism disorder, such as diabetic cardiomyopathy, autophagic function is often impaired, leading to exacerbated lipotoxicity and glucotoxicity [37,38], which, in turn, induce oxidative stress and endoplasmic reticulum (ER) stress, thereby activating autophagic programs. Both reactive oxygen species (ROS) and the ER unfolded protein response (UPR) can activate autophagy as a compensatory clearance mechanism. However, excessive stress can lead to overactivation of autophagy or lysosomal dysfunction, thereby inducing apoptosis or necrosis [39]. Recent findings indicate that angiotensin II induces autophagy via the angiotensin II type 1 receptor, promoting cardiomyocyte loss and playing a critical role in the progression of hypertrophy and heart failure [40].

### 2.3 The “Double-Edged Sword” Role of Autophagy in Cardiac Diseases

Studies have demonstrated that, in the early stages of pressure overload-induced cardiac hypertrophy, a moderate upregulation of autophagy exerts a compensatory protective effect. However, as the condition progresses to the decompensated phase and heart failure, this effect is often accompanied by impaired autophagic flux (*e*.*g*., decreased lysosomal degradation capacity), leading to autophagosome accumulation and exacerbated cellular injury [41]. Research indicates that dysfunction of the autophagy regulators, such as PTEN-induced putative kinase 1 (PINK1)/Parkin and Bcl2-interacting protein 3 (BNIP3)/NIP3-like protein X (NIX), results in the accumulation of dysfunctional mitochondria and impaired myocardial energy supply, thereby driving the progression of heart failure [42]. Severe coronary artery stenosis or occlusion induces myocardial ischemia and mitochondrial damage, activating autophagic processes that degrade damaged mitochondrial components, which enter the TCA cycle to generate ATP, thereby providing energy to cells and exerting a protective effect [43]. Conversely, during myocardial ischemia–reperfusion, excessive autophagic activation, together with oxidative stress bursts and calcium overload, may promote cell death [44]. Therefore, targeting the regulatory nodes of excessive autophagy may become a new therapeutic target for heart disease. In pressure overload-induced heart failure, autophagy is upregulated to maintain cellular homeostasis, whereas in age-related heart failure, autophagic function declines, leading to the accumulation of aberrant proteins and organelle dysfunction [45].

Recent studies have confirmed that mitophagy is a specialized process that recognizes and clears dysfunctional mitochondria, representing a core mechanism of mitochondrial quality control [46]. The most canonical pathway involved is the *PINK1*/*Parkin* axis. Upon loss of mitochondrial membrane potential, *PINK1* stabilizes on the outer mitochondrial membrane and recruits Parkin, which subsequently ubiquitinates mitochondrial surface proteins, facilitating the formation of a protein complex that initiates autophagic elimination [47]. In addition, outer mitochondrial membrane receptors such as *FUNDC1* can directly respond to stressors, including hypoxia, via phosphorylation-dependent regulation (*e*.*g*., modulation by kinases such as *ULK1* and *CK2*), thereby initiating mitophagy independently of the PINK1/Parkin pathway. Mitophagy relies on specific receptor proteins to achieve precise recognition. These receptors, including *FUNDC1*, *OPTN*, and *NDP52*, anchor to damaged mitochondria at one end and, via their LC3-interacting region (LIR), bind to *LC3* proteins on the autophagosomal membrane, thereby selectively enclosing mitochondria within autophagosomes for clearance [48,49]. Newly identified receptors such as *BNIP3*, *NIX*, and *FUNDC1*, along with the interplay between formin 1 and mitofusin 2, collectively regulate mitophagy and oxidative stress [50]. Under energy stress, AMPK directly phosphorylates ULK1 to activate autophagy while concurrently inhibiting the mechanistic target of rapamycin complex 1 (mTORC1); under nutrient-rich conditions, mTORC1 suppresses ULK1. Research indicates that AMPK activation confers protective effects during myocardial ischemia, whereas chronic overactivation may lead to detrimental outcomes. Transcription factor EB (TFEB), which is regulated by mTORC1, translocates from the cytosol to the nucleus in response to lysosomal dysfunction, activating the transcription of target genes that induce lysosomal biogenesis, enhance autophagic flux, and improve cardiac function. Macroautophagy provides fundamental support for survival and metabolic regulation under basal conditions in the heart. Mitophagy continuously eliminates aged or mildly damaged mitochondria, preventing excessive ROS production and the leakage of pro-apoptotic factors, both of which are essential for maintaining normal cardiomyocyte function [51]. Appropriate levels of mitophagy confer protection to cardiomyocytes, whereas either excessive or insufficient mitophagy is detrimental. In myocardial ischemia–reperfusion injury, moderate mitophagy facilitates the removal of damaged mitochondria and suppresses overactivation of deleterious pathways such as the NLRP3 inflammasome, thereby protecting cardiomyocytes. However, this process must be precisely regulated, as either excessive or insufficient mitophagy can exacerbate injury [52]. In the cardiovascular system, macroautophagy functions as a fundamental metabolic regulatory network in response to integrated stress, whereas mitophagy serves as a specialized quality control system targeting mitochondria. Several cardioprotective agents, such as citronellal [53] and astragaloside IV [54], act on the AMPK–mTOR pathway to modulate macroautophagic flux and on the PINK1/Parkin/FUNDC1 pathway to initiate mitophagy. The coordinated actions of both pathways collectively preserve cardiomyocyte homeostasis.

### 2.4 Autophagy and the PCD Pathway Have a Complex Bidirectional Regulatory Relationship

Basal autophagy can alleviate oxidative stress-induced damage by clearing damaged organelles, misfolded proteins, excess iron ions, and lipid peroxides. Basal autophagy also stabilizes key ferroptosis-related proteins solute carrier family 7 member 11 (SLC7A11) and glutathione peroxidase 4 (GPX4), thereby inhibiting apoptosis, ferroptosis, necroptosis, and pyroptosis [55]. Additionally, autophagy can degrade RIPK3 and inflammasome components, thereby limiting excessive inflammatory damage and exerting a pro-survival function. In contrast, excessively activated autophagy under stress conditions degrades anti-apoptotic proteins and key survival factors, inducing autophagic cell death and compensating for apoptotic pathways [56]. At the molecular level, core autophagy molecules ATG5/7 and Beclin 1 can bind to Bcl-2 and release BAX/BAK to initiate apoptosis. Conversely, activated caspase-3/8 can cleave Beclin 1, which promotes the cleavage of autophagy vesicle, thereby inhibiting autophagy. These two processes form a tight negative feedback regulatory loop that collectively maintains cellular homeostasis [57].

### 2.5 Prospects and Challenges of Targeted Autophagy Approaches

Targeting autophagy offers a novel therapeutic strategy for CVD, yet the process is also highly complex. Previous studies have demonstrated that several clinically used agents modulate cardiomyocyte autophagy and exert therapeutic effects by either inducing or inhibiting autophagy. Autophagy agonists include drugs for cardiovascular disorders (*e*.*g*., verapamil, amiodarone, metoprolol, propranolol, clonidine, digoxin, dronedarone, isoproterenol, norepinephrine, ivabradine, nifedipine, olmesartan, rapamycin, statins), antidiabetic medications (*e*.*g*., metformin, sodium–glucose cotransporter 2 (SGLT2) inhibitors), and anti-tumor agents (*e*.*g*., doxorubicin, bortezomib, tamoxifen). Meanwhile, autophagy inhibitors include paclitaxel (used to prevent in-stent restenosis) and ranolazine (used to treat stable angina and arrhythmia), among others [58]. Ponatinib, one of the most potent tyrosine kinase inhibitors for chronic myeloid leukemia, suppresses autophagosome formation and the expression of *LC3-II and p62* in cardiomyocytes, thereby blocking autophagic flux and inducing severe cardiotoxicity, manifested as vascular occlusion or thrombotic events [59]. Currently, intensive research is focused on the activation of autophagy by mTOR inhibitors such as rapamycin, the blockade of late-stage autophagy by lysosomal inhibitors including hydroxychloroquine, and the development of more specific modulators such as TFEB agonists and receptor-selective autophagy regulators [60]. These investigations are expected to yield major breakthroughs in this field.

The primary challenge for targeted therapy lies in achieving precise intervention with cell type-specificity, disease stage-specificity, and autophagy process-specificity. For instance, enhancing regulation of autophagic flux during the early stages of heart failure may facilitate recovery of cardiac function, whereas in advanced heart failure, restoring lysosomal function may be more critical than simply enhancing the initiation of autophagy [61]. Emerging technologies for monitoring autophagic flux (*e*.*g*., *mRFP–GFP–LC3* dual-fluorescence adenovirus) and gene-editing tools are laying the foundation for more precise research and intervention. Modest inhibition of autophagy during the reperfusion phase, whether via pharmacological agents or genetic approaches, has been shown to reduce infarct size. Activating autophagy (*e*.*g*., through rapamycin or caloric restriction mimetics) can improve cardiac function in aged individuals [62]. Resveratrol blocks excessive autophagy/mitophagy by inhibiting *LC3/ATG5/p62*-mediated autophagy and *PINK1/Parkin*-regulated mitophagy through the sirtuin 1/peroxisome proliferator-activated receptor gamma coactivator-1 alpha (SIRT1/PGC-1α) and TNF/NF-κB/JNK pathways, and reduces autophagic flux by inactivating the NF-κB pathway [63].

## 3. Research Progress on Molecular Mechanisms and Therapeutic Targets of Cardiomyocyte Apoptosis

Recent research indicates that the regulatory mechanisms of cardiomyocyte apoptosis have evolved from a relatively simple pathway model to a complex system integrating multiple signaling networks, epigenetic regulation, organelle interactions, and the convergence of novel cell death modalities.

### 3.1 “Apoptosis” Concept

Apoptosis refers to a process of cell death triggered by internal and external factors that activate a pre-existing death program within the cell. Apoptosis is a gene-regulated, active process, and the typical apoptotic cascade involves a series of proteolytic activations and signal transduction events mediated by cysteine–aspartic acid proteases (caspases) [64]. Apoptosis is closely related to embryonic development, organogenesis, tissue differentiation, and repair. To meet the needs of development or tissue renewal, cells in the body undergo apoptosis at specific times; for example, during development of the lens of the eye [65] and maturation of T lymphocytes in the thymus [66,67], thereby ensuring normal individual development and maintaining physiological function. As terminally differentiated cells, cardiomyocytes undergo irreversible loss, a core contributor to the deterioration of cardiac function. Apoptosis is a key pathological process in the progression of heart diseases such as heart failure, myocardial infarction (MI), and cardiomyopathy.

### 3.2 Major Signaling Pathways of Cardiomyocyte Apoptosis

The major signaling pathways of cardiomyocyte apoptosis include the mitochondrial-dependent apoptotic pathway (intrinsic pathway) and the death receptor pathway (extrinsic pathway) [68], as well as ER stress, non-coding RNA regulation, and epigenetic regulation [69].

**Mitochondrial (intrinsic) pathway:** Mitochondrial outer membrane permeabilization (MOMP) leads to the release of cytochrome c, which forms an apoptosome with *Apaf-1* and activates caspase-9. The Bcl-2 protein family acts as the gatekeeper at the onset of apoptosis. When the expression levels of anti-apoptotic proteins (such as Bcl-2, Bcl-xL, and Mcl-1) decrease or when pro-apoptotic proteins (such as Bax, Bak, Bim, and p53 upregulated modulator of apoptosis [PUMA]) are upregulated, the apoptotic program is initiated. Post-translational modifications such as phosphorylation and ubiquitination precisely regulate the activity and stability of Bcl-2 family proteins. Mitochondrial proteins, such as hexokinase-II, bind mitochondria to form anti-apoptotic complexes that dissociate during heart failure. The dynamic balance of Bcl-2 family proteins plays a crucial role in the apoptotic program. In addition to the inhibitory effect of anti-apoptotic Bcl-2 family members, Bax/Bak and Bcl-2/Bcl-xL also exert context-dependent regulatory effects. Recent studies have found that only BH3 proteins, such as Bim, Puma, and Noxa, induce apoptosis under stress conditions. Bim is upregulated in pressure-overload-induced heart failure; therefore, inhibiting Bim is considered an important therapeutic target [70,71]. The composition and regulation of MOMP have been increasingly clarified over the past decade. Studies have shown that Bax/Bak oligomerization forms MOMP and cooperates with the mitochondrial permeability transition pore (mPTP) to promote apoptosis. Meanwhile, inhibiting the mPTP opening can reduce apoptosis during ischemia–reperfusion [72]. Animal experiments have shown that Nrf3 promotes damage-induced cardiomyocyte apoptosis and worsens cardiac function by increasing mitochondrial ROS production through inhibition of Pitx2 expression. Therefore, targeting the Nrf3–Pitx2–mitochondrial ROS signaling axis may represent a novel therapeutic approach for MI [73].

**Death receptor (extrinsic) pathway:** The death receptor pathway is initiated by the binding of ligands such as tumor necrosis factor-alpha (TNF-α) and FasL to death receptors on the cell membrane, recruiting Fas-associated death domain protein (FADD) and activating caspases-8 and -10. In the myocardium, death receptor signaling can also trigger inflammatory and survival signals, with the outcome depending on the cellular microenvironment. The Fas/FasL, TNF-α/tumor necrosis factor receptor 1 (TNFR1), and TNF-related apoptosis-inducing ligand 1/death receptor 4/5 systems are activated in heart failure, promoting apoptosis. In patients with heart failure, myocardial expression of FADD and caspase-8 is significantly upregulated and negatively correlated with left ventricular ejection fraction [74]. Notably, this pathway interacts with the mitochondrial pathway, as caspase-8 can cleave Bid to generate tBid, thereby amplifying apoptotic signals [75].

**ER stress pathway:** The ER is an independent metabolic compartment and the primary site of protein and lipid synthesis, as well as calcium storage, in eukaryotic cells. Three transmembrane proteins on the ER membrane, protein kinase R-like ER kinase (PERK), inositol-requiring enzyme 1α (IRE1α), and activating transcription factor 6 (ATF6), act as the three main transducers of the UPR. Under persistent stress, these transducers directly induce apoptosis through mechanisms such as upregulation of C/EBP homologous protein (CHOP) and calcium release. Chronic or severe ER stress induces apoptosis through the CHOP, caspase-12, and c-Jun N-terminal kinase (JNK) pathways. Studies have shown that brain and muscle ARNT-like 1 (Bmal1) participates in the development of dilated cardiomyopathy through Bcl-2/inositol trisphosphate receptor -mediated mitochondrial calcium overload [76]. In diabetic cardiomyopathy, high glucose levels promote cardiomyocyte apoptosis via the IRE1α–X-box binding protein 1–CHOP axis, whereas IRE1α inhibitors can significantly improve cardiac function. The UPR is closely linked to many human diseases, and the development of targeted drugs remains a long and challenging task.

**Regulation by non-coding RNAs (ncRNAs):** Notably, ncRNAs, including circular RNAs (circRNAs), long non-coding RNAs (lncRNAs), and microRNAs (miRNAs), are unique RNA molecules widely identified in the eukaryotic genome. ncRNAs finely regulate apoptosis through multiple mechanisms, the most important of which is the competitive endogenous RNA (ceRNA) mechanism, which acts as a “molecular sponge” to bind miRNAs, thereby relieving miRNA-mediated inhibition of target genes and affecting the expression of apoptosis-related proteins [77]. Meanwhile, lncRNAs include *H19*, *GAS5*, and *PITAR*. Importantly, *PITAR* inhibits apoptosis through the *TRIM28/p53* pathway. Moreover, studies have found that lncRNA *H19* can function by adsorbing miR-29a-3p. Under normal circumstances, miR-29a-3p inhibits the expression of suppressor of cytokine signaling 3 (SOCS3), a signaling molecule involved in cell survival. H19 adsorbs miR-29a-3p via the “sponge” effect, relieving its inhibition of SOCS3 and enhancing cell viability and inhibiting apoptosis [78]. Furthermore, lncRNA *GAS5* acts as a ceRNA to adsorb miR-21 through the *miR-21*–*PTEN* axis, thereby upregulating expression of its target gene *PTEN*, inhibiting phosphorylation (*i*.*e*., activation) of protein kinase B (AKT), and altering the ratio of apoptosis-related proteins such as Bax and Bcl-2, ultimately promoting neuronal apoptosis [79]. The lncRNA *GAS5* can also act as a ceRNA, binding *miR-29b-3p* through the *miR-29b-3p*–*signal transducer and activator of transcription 3* (*STAT3*) axis, thereby upregulating STAT3 expression and regulating downstream apoptosis-related molecules such as Bax and caspase-3, thereby promoting apoptosis [80]. The lncRNA *PITAR* can directly bind to *TRIM28* mRNA and enhance its stability, thereby increasing TRIM28 protein levels. Therefore, *PITAR* indirectly promotes the degradation and inactivation of p53 by stabilizing *TRIM28* mRNA, thereby inhibiting apoptosis and promoting tumor growth [81]. Meanwhile, miRNAs inhibit the translation of target gene mRNA or promote mRNA degradation by binding to the 3’ untranslated region (UTR) of the target mRNAs. Additionally, miRNA-541-3p can directly negatively regulate the target gene *Rac2*. Downregulation of the target gene *Rac2* activates the TNF receptor superfamily member 10B (TNFRSF10B)-mediated signaling pathway, inducing necroptosis. Meanwhile, miRNAs such as miR-34a, miR-15, and miR-320 promote apoptosis in myocardial ischemia–reperfusion injury, whereas miR-21, miR-24, and others inhibit apoptosis and play protective roles in cardiomyocytes [82]. It has been shown that miR-34a promotes apoptosis by upregulating sirtuin 1 and Bcl-2 [83]. In summary, ncRNAs constitute a complex regulatory network that can act as a decoy to bind miRNAs or directly regulate the stability of key proteins, thereby precisely controlling the apoptotic switch at the post-transcriptional level. Histone deacetylases and the NOD-like receptor protein 3 (NLRP3) inflammasome are also involved in regulating apoptosis [84,85].

### 3.3 The Crosstalk Between Apoptosis and Other PCD Pathways

The death receptor pathway and the mitochondrial pathway are tightly coupled via the core molecule caspase. (1) Upon activation, extrinsic death receptors (*e*.*g*., Fas and TNFR1) activate caspase-8, which serves as a core molecular switch connecting apoptosis, necroptosis, and pyroptosis [86,87]. Activated caspase-8 cleaves the BH3-only protein Bid, generating its active form, tBid, which then translocates to mitochondria, disrupts mitochondrial membrane stability, and promotes cytochrome c release, thereby amplifying the intrinsic apoptotic signal and establishing a cascade-based crosstalk between the two pathways [87]. Concurrently, Bcl-2 family proteins function as a central regulatory hub: these proteins both regulate MOMP and bind to the key autophagy protein Beclin 1, directly mediating bidirectional regulation between apoptosis and autophagy [88]. (2) The core process of apoptosis relies on cascade activation of the caspase family and regulation by Bcl-2 family proteins, with caspase-8 serving as a central hub of crosstalk. Under normal conditions, caspase-8 activates caspase-3/7 to execute apoptosis. However, when caspase-8 activity is inhibited, its suppression of receptor-interacting protein kinase 1/receptor-interacting protein kinase 3 (RIPK1/RIPK3) cleavage is relieved, leading to RIPK3-dependent phosphorylation of mixed lineage kinase domain-like protein (MLKL), which subsequently triggers necroptosis. This establishes bidirectional antagonistic regulation between apoptosis and necroptosis. Furthermore, tBid generated by caspase-8-mediated cleavage of Bid can integrate into mitochondria and initiate the mitochondrial apoptotic pathway [89].

### 3.4 Translational Perspectives on Therapeutic Targeting of Cardiomyocyte Apoptosis

Novel strategies for targeted intervention in cardiomyocyte apoptosis are currently under investigation. These include small-molecule inhibitors of the pro-apoptotic protein Bax, BH3 mimetics that selectively inhibit anti-apoptotic proteins, and inhibitors of dynamin-related protein 1, such as mitochondrial division inhibitor 1, which mitigate mitochondrial fission. Additional approaches involve compounds that enhance mitophagy, inhibitors of the NLRP3 inflammasome, and activators of AMPK and sirtuins [90]. Epigenetic intervention-based therapies, such as miRNA-based therapeutics (*e*.*g*., anti-miR-34a), have demonstrated potential for clinical translation [91]. Apoptotic bodies have been shown to be cleared by phagocytes and, under specific conditions, to influence the function of neighboring cells and promote inflammation through the release of bioactive microvesicles [92]. Future directions include the development of more cardio-specific delivery systems, the utilization of organoids and artificial intelligence for compound screening, and the exploration of crosstalk between apoptosis and other forms of RCD, such as ferroptosis and pyroptosis, to identify novel therapeutic targets and develop effective inhibitors.

## 4. Molecular Mechanisms and Intervention Strategies for Cardiomyocyte Necrosis

### 4.1 Novel Concepts of Cardiomyocyte Necrosis

Cardiomyocyte necrosis is an important mechanism underlying the occurrence and development of various heart diseases. The extent, duration, and dynamics of cardiomyocyte necrosis vary significantly among different cardiac diseases. For example, MI is characterized by the death of large numbers of myocardial cells within 24 hours of ischemia. In contrast, heart failure leads to prolonged myocardial cell death lasting months to years [93]. Consequently, understanding of cardiomyocyte necrosis has evolved from the traditional view that necrosis is a passive and uncontrollable process to the contemporary recognition that necrosis can also be regulated. In the past, cardiomyocyte necrosis was considered to be ACD induced by ischemia and hypoxia, pathologically manifesting as coagulative necrosis characterized by organelle swelling, membrane rupture, and leakage of cellular contents, which, in turn, triggers a robust inflammatory response. A notable breakthrough in recent years has been the discovery that signaling pathways also regulate necrosis [94]. Therefore, necrosis is no longer viewed as an inevitable consequence of ischemia, but rather as a product of specific signaling cascades. Studies have revealed that even under conditions of calcium overload or ROS stimulation, cells do not undergo immediate rupture; instead, the cells progress toward death through distinct molecular pathways. The Nomenclature Committee on Cell Death has clearly defined ACD as a process resulting from extreme physical or chemical insults (such as burns or strong acids). In contrast, RCD encompasses forms of necrosis driven by well-defined molecular mechanisms, including necroptosis, ferroptosis, and pyroptosis [95]. In cardiomyocytes, the core region of an ischemic insult typically exhibits a mixture of ACD and RCD, whereas the ischemic penumbra (the region subjected to reperfusion) predominantly undergoes regulated necrosis. With this conceptual shift, clinical strategies for managing myocardial necrosis have undergone a fundamental transformation. Current approaches aim to block transmission of necrotic signals along these pathways, potentially delaying or preventing cardiomyocyte rupture. In summary, the new concept posits that cardiomyocyte necrosis, particularly under ischemia–reperfusion conditions, is a form of PCD tightly controlled by signaling pathways. This discovery provides new theoretical foundations and potential therapeutic targets for the precision treatment of cardiac diseases. Targeting these pathways (*e*.*g*., RIPK3–MLKL and GPX4–ferroptosis) has emerged as a new frontier in the treatment of CVD [96].

### 4.2 Major Regulatory Pathways of Cardiomyocyte Necrosis

The pathways involved in cardiomyocyte necrosis include autosis, necroptosis, ferroptosis, pyroptosis, and necrosis mediated by mPTP, NECSO, cuproptosis, and disulfidptosis, as well as regulated signaling pathways. These interconnected pathways form a complex and interactive network that regulates cell necrosis [97].

#### 4.2.1 Autosis: Regulation and Targeted Intervention

Autosis is a distinctive form of PCD that strictly depends on both the core autophagic machinery and Na^+^/K^+^-ATPase activity. Morphologically, autosis is characterized by dilation of the perinuclear space, bell-shaped nuclear shrinkage, and mild organelle swelling. Autosis represents a subtype of autophagy-dependent cell death [98]. In contrast, conventional hyperactive autophagy typically manifests as extensive accumulation of autophagic vacuoles, widespread engulfment and degradation of organelles, and eventual cell disintegration or necrosis-like changes, without reliance on Na^+^/K^+^-ATPase, which is a key distinction from autosis. Meanwhile, autosis cannot be reversed by inhibitors of apoptosis or necrosis, whereas pharmacological inhibition of Na^+^/K^+^-ATPase (*e*.*g*., by cardiac glycosides or knockdown of the α1 subunit) completely blocks autosis without affecting conventional autophagic cell death. Autosis is finely regulated by multiple signaling pathways, with the core regulatory axis being the class III phosphatidylinositol 3-kinase (PI3K)–Beclin 1–Na^+^/K^+^-ATPase complex. Class III PI3K (VPS34) forms a complex with Beclin 1 to initiate autophagy, and direct binding of Beclin 1 to the α subunit of Na^+^/K^+^-ATPase constitutes a specific molecular event in autosis [99]. The upstream regulatory pathways include: (1) the AMPK–mTOR pathway, in which AMPK inhibits the mTORC1 under energy deprivation, activates the ULK1 complex, and promotes assembly of the core complex to initiate autosis [100]; (2) the TFEB pathway, in which TFEB translocates to the nucleus during myocardial ischemia–reperfusion, upregulates autophagy- and lysosome-related genes, and exacerbates autosis; (3) oxidative stress (ROS), in which high levels of ROS activate autosis via the hypoxia-inducible factor 1 alpha (HIF-1α)–TFEB axis, whereas low levels of ROS suppress this process [101].

In myocardial ischemia–reperfusion injury, moderate autophagy is protective during the ischemic phase, whereas excessive autophagic activation during the reperfusion phase triggers autosis, leading to extensive cardiomyocyte death, enlarged infarct size, and worsened cardiac function. The underlying mechanisms are closely associated with TFEB-mediated upregulation of autophagic genes, enhanced Beclin 1–Na^+^/K^+^-ATPase interaction, and ROS cascade amplification. Selective inhibition of autosis confers substantial cardioprotection [98,99]. Currently, agents targeting autosis include inhibitors such as cardiac glycosides (*e*.*g*., digoxin, which blocks the binding between the Na^+^/K^+^-ATPase α subunit and Beclin 1; insulin-like growth factor-1 decreases Beclin 1–Na^+^/K^+^-ATPase interaction and inhibits autosis) [100,101], the sodium–glucose cotransporter 2 (SGLT2) inhibitor empagliflozin (which suppresses TFEB nuclear translocation, downregulates autophagy-related genes, and reduces myocardial autosis) [102], and 3-methyladenine (3-MA), which inhibits class III PI3K and broadly blocks autophagy initiation. Agonists include *Tat-BECN1* (which targets and activates *Beclin 1*, enhances *Beclin 1*–Na^+^/K^+^-ATPase interaction, and potently induces autosis) and 3,4-dimethoxychalcone (which activates TFEB and promotes autophagic flux and autosis) [103,104].

#### 4.2.2 Necroptosis

Activation of death receptors, such as tumor necrosis factor receptor 1, initiates necroptosis. RIPK1 and RIPK3 are the most critical intracellular signaling factors mediating necroptosis. Upon binding of Fas (death receptor) to the associated ligand *FasL*, the RIPK1/RIPK3/MLKL complex is formed. Phosphorylated MLKL oligomerizes at the cardiomyocyte membrane, forming plasma membrane pores that lead to loss of membrane integrity and cell death. RIPK3 deficiency has been shown to reduce myocardial infarct size [105]. Caspase-8 acts as a molecular switch for apoptosis, necroptosis, and pyroptosis [106]. Elevated caspase-8 activity induces apoptosis and pyroptosis, whereas inhibition of caspase-8 initiates necroptosis. In ischemic myocardium, downregulation of cellular inhibitor of apoptosis protein 1/2 leads to insufficient caspase-8 inhibition, thereby promoting necroptosis [107,108]. Animal experiments showed that xanthosine (XTS), an intermediate of purine metabolism, improved cardiac remodeling and function after ischemia–reperfusion injury by inhibiting ferroptosis and reducing mitochondrial ROS production. This finding contributes to research on the development of XTS as a new adjuvant formulation for patients with acute MI [109].

#### 4.2.3 Ferroptosis

Ferroptosis is a form of regulated necrosis driven by iron-dependent accumulation of lipid peroxides, ultimately leading to cell membrane rupture. Iron is taken up via transferrin receptors and stored in ferritin. Ferritinophagy degrades ferritin, releasing Fe^2+^, which converts low-activity H_2_O_2_ into highly reactive hydroxyl radicals via the Fenton reaction, initiating a chain reaction of lipid peroxidation. In the presence of iron and ROS, polyunsaturated fatty acid phospholipids are oxidized to lipid hydroperoxides and further converted into deleterious end products (*e*.*g*., 4-hydroxynonenal), thereby compromising membrane integrity and function. The core defense mechanism involves GPX4 [110]. Cardiac-specific GPX4 knockout mice spontaneously develop heart failure, which is significantly ameliorated by the ferroptosis inhibitor ferrostatin-1. In cardiomyopathy and acute myocardial ischemia–reperfusion injury, massive ROS bursts and iron metabolism dysregulation can trigger ferroptosis, exacerbating tissue damage. Esterification of long-chain polyunsaturated fatty acids, mediated by acyl-CoA synthetase long-chain family member 4, constitutes an essential lipid synthesis step for ferroptosis in cardiomyocytes and represents a potential intervention target [111]. Ferroptosis exhibits extensive pathway crosstalk with other forms of PCD [112]. ROS, which serves as a common stress signal, coordinate regulation of lipid peroxidation, mitochondrial apoptosis, and NLRP3 inflammasome activation, thereby linking multiple cell death pathways [113]. As a central regulatory molecule, p53 not only suppresses SLC7A11 to promote ferroptosis but also mediates BAX signaling to induce apoptosis, thereby positioning this protein as a key hub of crosstalk. Dysregulated iron metabolism exacerbates RIPK1 phosphorylation, mediating the intersection of ferroptosis and necroptosis. PANoptosis, orchestrated by the PANoptosome complex and involving core molecules such as RIPK3 and caspase 8, synergistically triggers multiple modes of cell death in response to cellular stress and infectious stimuli [114]. These various cell death pathways are mutually regulated and tightly interconnected; the homeostatic imbalance of these pathways contributes to the pathogenesis and progression of multiple diseases, including inflammation and cancer, thereby offering new directions for research into disease mechanisms and targeted interventions.

#### 4.2.4 Pyroptosis

Upon stimulation by signals such as bacteria or viruses, intracellular pattern-recognition receptors initiate a recognition-signaling cascade. This leads to the formation of a multiprotein complex via the adaptor protein apoptosis-associated speck-like protein containing a CARD and pro-caspase-1, thereby activating caspase-1. Meanwhile, activated caspase-1, together with activated caspases-4, -5, and -11, regulates inflammation through two pathways: first, by cleaving gasdermin D (GSDMD) to release its N-terminal active domain, which forms pores in the cell membrane, causing cell rupture and content release and thereby directly inducing inflammation; second, by cleaving pro-interleukin (IL)-1β and pro-IL-18, converting these proteins into the associated active forms (IL-1β and IL-18), which are secreted extracellularly to recruit inflammatory cells and amplify the inflammatory response.

Furthermore, granzyme-mediated pathways also contribute to pyroptotic cell death. Granzyme A (GZMA), the most abundant serine protease in the granzyme family, is traditionally considered a mediator of cell death. GZMA, originating from cytotoxic lymphocytes, rapidly activates caspase-3 and cleaves gasdermin B (GSDMB) in target cells, generating N-terminal GSDMB fragments that form pores in the cell membrane and promote pyroptosis. In viral myocarditis, cardiomyocytes release IL-1β/IL-18, amplifying inflammation and leading to cell swelling and rupture [115,116]. Caspase-8 can cleave GSDME, triggering pyroptosis and converting apoptosis into inflammatory cell death. Necroptosis depends on the RIPK1–RIPK3–MLKL signaling axis. When caspase-8 activity is inhibited, death receptor signals shift to activate RIPK3, initiating non-caspase-dependent programmed necrosis. Meanwhile, activated RIPK3 can promote pyroptosis by regulating inflammasome assembly and facilitating caspase-1 activation. Pyroptosis is a form of inflammatory PCD that relies on inflammasome-mediated activation of caspase-1/4/5/11, which cleave GSDMD, forming membrane pores that lead to cell lysis. Multiple crosstalk mechanisms include caspase-8 directly cleaving GSDMD to initiate pyroptosis; RIPK3 regulating NLRP3 inflammasome assembly to promote caspase-1 activation and GSDMD cleavage, thereby linking necroptosis and pyroptosis; and caspase-3 cleaving GSDME to induce pyroptosis, thereby converting apoptotic cells into an inflammatory death mode under specific stress conditions and amplifying immune responses [117].

Pyroptosis is an inflammatory form of PCD mediated by the GSDM family. Pyroptosis engages in extensive molecular crosstalk with apoptosis, necroptosis, and ferroptosis, collectively forming a finely regulated RCD network. A central node of this network is the caspase family. The apoptotic executors caspase-3/7 can cleave GSDMD, thereby converting apoptotic signals into inflammatory pyroptosis. Caspase-8 exerts dual regulatory functions: Caspase-8 mediates apoptosis, cleaves GSDMD/GSDMC to trigger pyroptosis, and inhibits the RIPK1–RIPK3–MLKL necroptotic axis [118]. In necroptosis, MLKL forms pores, leading to potassium efflux, which activates the NLRP3 inflammasome and subsequently induces pyroptosis via the caspase-1–GSDMD pathway [119]. Ferroptosis, driven by ROS accumulation and lipid peroxidation, activates NLRP3, thereby establishing a positive feedback loop (ROS–NLRP3) with pyroptosis. Recent studies have identified the PANoptosome complex, which integrates signals from all three pathways. RIPK3 serves as a central hub, concurrently activating MLKL, Caspases, and GSDMD, culminating in PANoptosis, a cell death modality exhibiting features of pyroptosis, apoptosis, and necroptosis [120,121]. This intricate interplay among pathways ensures redundancy in cellular stress responses and provides multiple targetable nodes for treating inflammatory diseases and cancers.

#### 4.2.5 mPTP-Mediated Necrosis

The mPTP is considered a core mechanism of necrosis. Oxidative stress and calcium ion (Ca^2+^) overload trigger the mitochondrial permeability transition (MPT)-driven necrotic pathway. Conditions such as ischemia-reperfusion injury or exposure to certain toxins cause a substantial increase in cytosolic Ca^2+^. Mitochondria take up Ca^2+^ via the mitochondrial calcium uniporter (MCU), leading to Ca^2+^ overload in the mitochondrial matrix and a burst of ROS. During Ca^2+^ overload and oxidative stress, cyclophilin D (CypD) binds to pore structural proteins (adenine nucleotide translocase or F_0_F_1_-ATPase), inducing conformational changes. In conjunction with Ca^2+^ and ROS, this causes the instantaneous opening of the mPTP, which results in a dramatic increase in the permeability of the inner mitochondrial membrane to molecules smaller than 1.5 kDa (including protons, ions, and metabolites), leading to collapse of the mitochondrial membrane potential and uncoupling of oxidative phosphorylation, thereby preventing ATP synthesis by ATP synthase. Simultaneously, to maintain the membrane potential, cytosolic ATP drives the reverse operation of Na^+^/K^+^-ATPase and Ca^2+^-ATPase, consuming large amounts of ATP and causing a sharp decline in ATP levels. Collapse of membrane potential leads to excessive electron transport chain activity, generating more ROS [122,123]. High ROS levels further promote mPTP opening, creating a vicious cycle that results in mitochondrial swelling, outer membrane rupture, and cell death. CypD is the target of cyclosporine A (CsA). CsA inhibits mPTP opening by binding to CypD and preventing the interaction with pore components such as ATP synthase. Blocking MCU reduces mitochondrial Ca^2+^ uptake and delays mPTP opening.

#### 4.2.6 NECSO

Sodium ions, the most abundant cations in cells, play a crucial role in maintaining membrane potential, regulating intracellular osmotic pressure, and ensuring normal cellular function. NECSO represents a novel form of necrotic cell death characterized by sodium overload. While the underlying mechanisms remain incompletely defined, NECSO has been linked to the *TRPM4* gene [8].

Under stress conditions such as ischemia or organ failure, or upon exposure to the chemical agonist necrocide 1 (NC1), the TRPM4 channel is activated, mediating Na^+^ influx. This subsequently elevates mitochondrial Na^+^ and reduces Ca^2+^ via the Na^+^/Ca^2+^ exchanger, inhibiting oxidative phosphorylation and the TCA cycle. The ultimate consequences are inactivation of Na+/K+-ATPase, loss of ionic gradients, cellular swelling, and lysis [124]. TRPM4-mediated NECSO may play a significant role in cardiac diseases characterized by energy depletion. Recent studies have also identified that dihydropyridine-type calcium channel blockers and clotrimazole can inhibit the NC1-induced NECSO process. NC1 acts on the non-selective monovalent cation channel *TRPM4*, promoting sodium influx and triggering cell necrosis. Cells deficient in TRPM4 are resistant to NC1-induced NECSO. Due to structural differences in the transmembrane region, NC1 specifically activates human TRPM4 but not the murine ortholog [125].

#### 4.2.7 Cuproptosis

Cuproptosis is a form of cell death driven by intracellular copper accumulation, leading to instability of iron–sulfur clusters and aggregation of acylated proteins, culminating in oxidative stress-associated cell death. The trigger for cuproptosis is excessive accumulation of copper ions within mitochondria, where copper binds to lipoylated proteins, disrupting mitochondrial metabolic enzymes and inducing proteotoxic stress [126]. A recent study revealed that radiotherapy elevates mitochondrial copper levels by upregulating copper transporter 1 and depleting the mitochondrial copper-chelating protein glutathione, thereby inducing cuproptosis. Research demonstrates that cuproptosis is characterized by dependence on copper and mitochondrial respiration, distinguishing this process from established forms of apoptosis, necrosis, pyroptosis, and ferroptosis. Excessive copper induces cuproptosis by promoting aberrant oligomerization of copper-dependent lipoylated proteins in the TCA cycle and reducing levels of iron-sulfur cluster proteins [127]. Ferredoxin 1 facilitates lipoylation of dihydrolipoamide transacetylase by reducing Cu^2+^ to Cu^+^ and decreases iron-sulfur cluster proteins, thereby inducing cell death. Cuproptosis has been associated with the onset and progression of myocardial ischemia–reperfusion injury, heart failure, atherosclerosis, and cardiac arrhythmias. Copper chelators such as ammonium tetrathiomolybdate and D-penicillamine may alleviate these cardiovascular conditions by inhibiting cuproptosis pathways. Oxidative phosphorylation inhibitors may suppress cuproptosis by mitigating proteotoxic stress. Intracellular copper concentrations are maintained at very low levels through active homeostatic mechanisms; once this threshold is exceeded, copper becomes toxic and ultimately induces cell death [128].

#### 4.2.8 Disulfidptosis

Disulfidptosis is a novel form of RCD, distinct from apoptosis, ferroptosis, necroptosis, and cuproptosis. Disulfidptosis is driven by disulfide stress resulting from the intracellular accumulation of excess cystine mediated by SLC7A11. The core mechanism involves aberrant intracellular disulfide accumulation, leading to collapse of the actin cytoskeleton and subsequent cell death [129]. This disulfide stress is closely associated with CVDs, and has been demonstrated to exacerbate the progression of various cardiovascular conditions, including atherosclerosis, hypertension, myocardial ischemia–reperfusion injury, diabetic cardiomyopathy, cardiac hypertrophy, and heart failure [130,131]. The underlying mechanism may involve disulfide stress, a specific oxidative stress state arising from an imbalance between the intracellular glutaredoxin and thioredoxin antioxidant systems. Abnormal accumulation of disulfide bonds directly drives disulfidptosis and independently damages cardiomyocytes, endothelial cells, and fibroblasts, positioning this process as a central factor in cardiovascular pathology. Several key proteins in the regulatory pathways of disulfidptosis are emerging as promising therapeutic targets [132,133]. Studies have identified aberrant expression of disulfidptosis-related genes, including *ACTN4*, *MYH10*, *TLN1*, *DSTN*, and *NCKAP1*, in dilated cardiomyopathy, potentially contributing to disease progression by altering cytoskeletal dynamics and energy metabolism [134]. Research has also confirmed that genes, including *GYS1*, *MYH10*, *PDMIL1*, *SLC3A2*, and *CAPZB*, are associated with hypertrophic cardiomyopathy and may serve as markers of disulfidptosis [135].

#### 4.2.9 Crosstalk and Integrated Regulation Among Necrotic Pathways

RCD pathways do not function in isolation; instead, these pathways form a highly integrated crosstalk network through shared molecules, signaling hubs, and metabolic nodes that collectively determine cell fate and contribute to disease progression. Apoptosis and autophagic cell death are governed by the Bcl-2 family–Beclin 1 complex as a core molecular switch. The anti-apoptotic proteins Bcl-2 and Bcl-xL directly bind to Beclin 1 to inhibit the initiation of autophagy. Upon activation of the apoptotic pathway, caspases cleave Bcl-2, releasing Beclin 1 and enabling the dynamic transition between apoptosis and autophagy. The PI3K/AKT/mTOR and AMPK signaling axes, as well as the subcellular localization of p53, further modulate the balance between the two pathways, which play pivotal roles in energy stress and tumor drug resistance [136].

Necroptosis mediates membrane lysis via the RIPK1/RIPK3/MLKL pathway, whereas ferroptosis is characterized by GPX4 inactivation and lipid peroxidation. These two pathways are tightly coupled through mitochondrial ROS and CaMKII signaling. RIPK3 activation exacerbates oxidative stress and increases sensitivity to ferroptosis; in turn, mitochondrial damage induced by ferroptosis reversely activates RIPK3, thereby forming a negative feedback loop. Both pathways amplify inflammatory responses via the NLRP3 inflammasome and synergistically aggravate tissue injury during ischemia–reperfusion injury [136].

Pyroptosis is a proinflammatory cell death mode executed by caspase-1/4/5/11-mediated cleavage of GSDMD. PANoptosis integrates the core characteristics of pyroptosis, apoptosis, and necroptosis and is driven by the PANoptosome complex comprising NLRP3, RIPK1/3, caspase-8, and FADD. Caspase-8 activity determines whether apoptosis occurs or PANoptosis is initiated. Inflammatory factors and exosomes released during pyroptosis further amplify PANoptosis signaling, forming a proinflammatory positive feedback loop in infectious and inflammatory diseases [137].

Cuproptosis and ferroptosis are functionally coupled via the FDX1–GPX4 axis. FDX1 regulates copper ion reduction and mediates GPX4 degradation, and copper overload directly suppresses GPX4 activity. The two pathways share the cystine transporter SLC7A11/GSH/GPX4 metabolic axis, which jointly promotes oxidative stress and pathological protein aggregation in neurodegenerative diseases [138,139].

Disulfidptosis is induced by glucose starvation, NADPH depletion, and disulfide stress, with SLC7A11 acting as a critical switching node. Under glucose sufficiency, SLC7A11 maintains GSH synthesis to restrain ferroptosis; under glucose deprivation, this sufficiency triggers disulfidptosis, enabling flexible switching between the two death modes [140,141].

Sodium overload-induced cell death is initiated by TRPM4 channel-mediated Na^+^ influx and proceeds independently through disruption of mitochondrial energy metabolism. This process inhibits apoptosis and necroptosis through ATP depletion, exacerbates ROS accumulation, thereby increasing susceptibility to ferroptosis, and acts synergistically with multiple cell death pathways in ischemic injury [138].

The caspase family, ROS, and mitochondria constitute the core cross-pathway hubs. Caspase-8 and caspase-3 respectively dominate the conversion of cell death modes; ROS, as a universal signaling molecule, activates ferroptosis, necroptosis, and the NLRP3 inflammasome; mitochondria serve as a common target that simultaneously drives multiple cell death pathways [136]. Glucose metabolism, ion homeostasis, and redox balance provide the metabolic foundation for pathway crosstalk, and autophagy bidirectionally regulates distinct cell death patterns via ferritinophagy, mitophagy, and other mechanisms [136,138].

The above crosstalk network has important pathological significance in tumors, neurodegenerative diseases, ischemia–reperfusion injury, and inflammatory diseases. Targeting a single pathway is prone to therapeutic failure due to compensatory activation of other pathways. Combined intervention at key crosstalk nodes such as RIPK3–GPX4, SLC7A11–FDX1, and the PANoptosome, together with single-cell transcriptomics to precisely identify cell-specific death modes, has become a core direction for the development of novel therapeutic strategies [139,140,141]. The crosstalk among these programmed cell death signaling pathways is shown in Fig. 2.

**Fig. 2.**
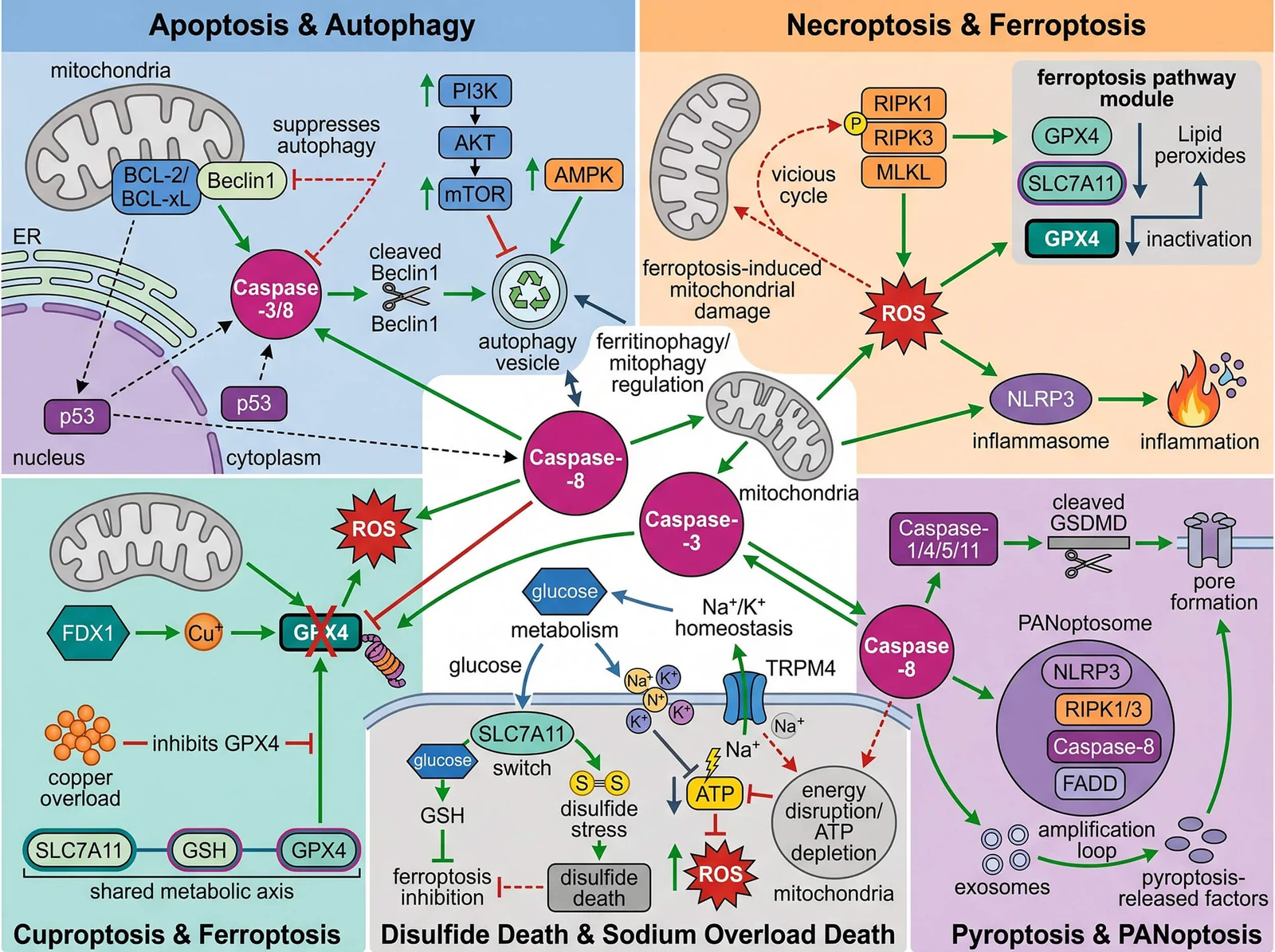
**Schematic representation of the crosstalk among selected PCD signaling pathways**. Reactive oxygen species (ROS) and members of the caspase family serve as central hubs connecting various PCD pathways. Red line segments represent inhibitory effects, and green arrows represent inductive or excitatory effects. Red dashed arrows indicate promoting effects. PCD, programmed cell death; GPX4, glutathione peroxidase 4; PI3K, phosphatidylinositol 3-kinase; AKT, Protein kinase B; AMPK, AMP-activated protein kinase; GSH, Glutathione; FADD, Fas-associated death domain protein. Fig. 2 was created with the assistance of software CorelDRAW Graphics Suite 2018.

#### 4.2.10 Research Progress on Targeted Interventions in Myocardial Cell Death Pathways

Recently, novel interventional agents targeting dual or triple pathways, including natural products, nanomedicines, RNA-based therapeutics, and metabolic modulators, have entered experimental research. Among these, various inhibitors targeting necroptosis-related pathways have emerged as a focus of research. Necroptosis inhibitors, such as RIPK1 inhibitors (*e*.*g*., necrostatin-1 and its derivatives), have demonstrated cardioprotective effects across multiple animal models [142]. In a porcine model of MI, Nec-1s reduced infarct size by up to 35%. The novel RIPK1 inhibitor Eclitasertib exhibited favorable tolerability in Phase I clinical trials, achieving >90% inhibition of RIPK1 phosphorylation at doses of at least 100 mg [143]. Furthermore, necrosulfonamide and novel compounds such as NSA-043 are currently in preclinical development. Z-DNA-binding protein 1 (ZBP1) is a core molecular driver of PANoptosis in cardiomyocytes. The small-molecule inhibitor MSB precisely blocks ZBP1, while GSK872 (an RIPK3 inhibitor) and NSA derivatives (targeting MLKL) have shown enhanced cardioprotective effects with reduced toxicity in models of diabetic cardiomyopathy and sepsis [144,145]. Ferroptosis inhibitors, including iron chelators such as deferoxamine, deferiprone, and novel oral iron chelators, attenuate ischemia–reperfusion-induced ferroptosis. Lipid antioxidants such as ferrostatin-1 and liproxstatin-1 directly neutralize lipid radicals; notably, liproxstatin-1 significantly ameliorates myocardial fibrosis and contractile dysfunction in models of diabetic cardiomyopathy. Studies indicate that ferroptosis is involved in ischemia–reperfusion-induced myocardial injury, drug-induced cardiomyopathy, diabetic cardiomyopathy, septic cardiomyopathy, as well as inflammation- and stress-induced myocardial damage. Activation of AMPK, PKC, ERK1/2, PI3K, and Akt protects against myocardial ferroptosis, whereas HO-1 inhibition alleviates ferroptosis [146]. Conversely, TLR4 activation and NF-κB activation promote myocardial ferroptosis. miR-450b-5p and miR-210-3p enhance cardiomyocyte tolerance to hypoxia/reoxygenation by inhibiting ferroptosis, whereas circ_0091761 and several miRNAs, including miR-214-3p and miR-199a-5p, exacerbate myocardial ferroptosis [53]. The roles of GSK-3β and NOS in regulating ferroptosis warrant further investigation, while activation of Nrf2 and STAT3 prevents ferroptosis. Recent studies have revealed a critical role for methyltransferase-like 3 (METTL3)-mediated m^6^A modification in ferroptosis following MI: METTL3 promotes GPX4 degradation via the autophagy-lysosome pathway by m^6^A-modifying *Dnajb1* mRNA and reducing its stability, suggesting that the METTL3–*Dnajb1* axis represents a novel anti-ferroptotic target [147]. ISRIB, a small-molecule ATF4 inhibitor, significantly attenuates ferroptosis and improves cardiac function by suppressing ATF4 expression [148]. Bardoxolone methyl, while activating Nrf2, also inhibits SLC7A11 and GPX4 expression, thereby inducing ferroptosis; the Phase III clinical trial on bardoxolone methyl was terminated due to adverse events including heart failure [149]. Dexmedetomidine inhibits ferroptosis via the Akt/GSK3β/Nrf2 axis, attenuating doxorubicin-induced cardiotoxicity without compromising its antitumor efficacy [150]. Moreover, direct knockdown of heme oxygenase-1 (HO-1) or administration of the HO-1 inhibitor ZnPP, which specifically inhibits HO-1 overexpression, has been shown to prevent ferroptosis [151].

The pyroptosis inhibitor MCC950 has been validated in animal models of heart failure and myocarditis [152,153], and inhibits interleukin-1β maturation and cardiomyocyte pyroptosis, thereby improving outcomes in septic cardiomyopathy [154,155]. MCC950 also suppresses activation of the NLRP3/ASC/caspase-1/GSDMD-N axis, reducing macrophage pyroptosis and IL-1β/IL-18 production in the aorta and cell lysates. Meanwhile, although INF150 protected macrophages, INF150 was unable to penetrate cardiomyocytes and exhibited no protective effect in an *ex vivo* perfused heart model [156]. In the context of heart failure with preserved ejection fraction (HFpEF), renal denervation reduces myocardial norepinephrine levels, inhibits activation of the ATP–P2X7–NLRP3 axis, and alleviates pyroptosis and diastolic dysfunction [157]. The GSDMD inhibitor disulfiram, which covalently modifies GSDMD and inhibits its pore-forming activity, has potential cardioprotective effects. The novel GSDMD inhibitor GI-Y1 specifically binds to the N-terminal domain of GSDMD and blocks oligomerization; the macrophage membrane-coated formulation of GI-Y1 (GI-Y1@MM-NPs) increases cardiac enrichment by 3.2-fold. The oral GSDMD inhibitor Pyrostat-HF has demonstrated a 43% reduction in pyroptosis markers and an 8.5% increase in left ventricular ejection fraction in a Phase IIb clinical study, with a Phase III trial imminent [158].

Among agents targeting the mPTP, the novel cyclophilin D inhibitor SLV-V166 exhibits high selectivity, along with cyclosporine A analogs. Furthermore, novel non-immunosuppressive cyclophilin D inhibitors (*e*.*g*., NIM811 and Alisporivir) avoid immunosuppressive side effects and offer improved clinical applicability. Bentamapimod dually inhibits p38 MAPK and CypD. The novel cardiac-homing nanomedicine H-FAME targets mitochondria, inhibits UCP2, and scavenges mtROS [159,160]. Among multi-target agents, the dihydropyrazole derivative compound 23a combines RIPK1 inhibition with potent antioxidant and anti-ferroptotic activities. ML355, an ALOX12 inhibitor, protects against ischemia–reperfusion injury by blocking the arachidonate 12-lipoxygenase (ALOX12) → neural precursor cell expressed developmentally downregulated 4 (NEDD4) → transforming growth factor-β-activated kinase 1 (TAK1) → AMPK (ALOX12 → NEDD4–TAK1 → AMPK) pathway [161]. Spermidine improves mitochondrial biogenesis via SIRT3 activation and alleviates antitumor agent-induced cardiotoxicity. The novel endogenous cardioprotective peptide S100A1 is downregulated in failing myocardium; supplementation with synthetic S100A1 improves contractile function, increases ATP production, exerts antiarrhythmic effects, and reduces inflammatory cytokine levels [162].

Ongoing research projects include a Phase I clinical study of Eclitasertib [143]; SIR9900 has completed a Phase I study [9]; investigation of the protective role of the integrated stress response inhibitor (ISRIB) in septic cardiomyopathy is advancing into large-animal models [8]; small-molecule inhibitors of METTL3 are in early-stage development, and *Dnajb1* gene therapy has shown preliminary efficacy [147]; next-generation NLRP3 inhibitors with high cardiomyocyte uptake are under development [4]. Inhibitors targeting the crosstalk axes of PCD signaling pathways, such as Sibiriline, Zharp1-163, and dual- or triple-node inhibitors, represent important directions for future research. From January 2024 to Jannuary 2026, a summary of novel experimental agents intervening in myocardial cell necrosis is presented in Table 1 (Ref. [142,144,145,146,158,160,161,163,164,165,166,167,168,169,170,171,172,173,174,175,176]).

**Table 1.** **Summary of newly developed formulations for cardiomyocyte necrosis intervention from January 2024 to January 2026**.

Targeted pathway	Formulation/drug name	Target and mechanism of action	Development stage	Core advantages	References
Necroptosis/panoptosis	MSB	Targets ZBP1, blocks ZBP1/RIPK3 panoptosome assembly, and inhibits the myocardial panoptosis cascade	Preclinical lead compound	Targets a novel panoptosis pathway; provides superior cardioprotection in myocardial ischemia–reperfusion injury compared with traditional RIPK1 inhibitors	[163]
	Sibiriline	Dual inhibition of RIPK1 kinase activity and clearance of lipid peroxide; synergistically blocks necroptosis and ferroptosis	Preclinical	Single-molecule dual-pathway regulation; synergistic anti-inflammatory and antioxidant cardioprotection	[164]
	Zharp1-163	Inhibits RIPK1, scavenges ROS, and downregulates lipid peroxidation; alleviates myocardial oxidative damage and ferroptosis	Preclinical	Combines anti-necrotic, antioxidant, and anti-inflammatory effects; applicable to multiple types of cardiomyopathy-related injury	[142]
	GSK872	Highly selective RIPK3 inhibitor that blocks downstream MLKL membrane pore formation; inhibits myocardial necroptosis	Preclinical	Low adverse effects; prominent efficacy in diabetic and septic cardiomyopathy	[144] [145]
Ferroptosis	Alda-1	ALDH2-specific agonist that corrects the ALDH2*2 functional defect and downregulates ACSL4/TFRC; reduces iron accumulation and lipid peroxidation	Preclinical/Phase I preparation	Targets high-prevalence genetic subtypes in the Chinese population; regulates key upstream ferroptosis pathways	[165]
	FBS0701	Novel oral iron chelator that selectively chelates free iron ions; reduces myocardial iron overload and inhibits ferroptosis initiation	Preclinical	Strong cardiac tissue targeting and enrichment; reduces myocardial infarct size by 52%	[166] [146]
	UAMC-3203	Second-generation lipid antioxidant that directly neutralizes lipid radicals; stabilizes cellular lipid homeostasis	Preclinical	Significantly improved water solubility and *in vivo* stability compared with ferrostatin-1 and liproxstatin-1	[167]
	Ciclopirox olamine	Drug repurposing; combines iron chelation and ACSL4 inhibitory activity; blocks key drivers of ferroptosis	Phase I clinical	Well-established safety profile; short development cycle; applicable to anticancer drug-induced cardiac injury	[168]
	Protosappanin A (PrA)	Targets the ACSL4/FTH1 signaling axis, reduces lipid peroxidation; simultaneously inhibits myocardial fibrosis	Preclinical	Natural plant-derived active compound; low toxicity; suitable for long-term myocardial protection	[169]
	Salvianolic acid C	Activates the Nrf2/HO-1 antioxidant pathway and scavenges mitochondrial ROS; inhibits myocardial ferroptosis and oxidative stress	Preclinical	Derived from a traditional Chinese medicine monomer; multi-target regulation integrating antioxidant, anti-inflammatory, and anti-necrotic effects	[146]
Pyroptosis	GI-Y1	Specifically binds to the GSDMD-N-terminal domain, inhibits GSDMD oligomerization and membrane pore formation; blocks myocardial pyroptosis	Preclinical	High target selectivity; does not interfere with normal apoptotic physiological processes	[170]
	GI-Y1@MM-NPs	Macrophage membrane-modified nanoparticle delivery system; targets and enriches in myocardial tissue; releases GI-Y1 in a sustained manner	Preclinical	Cardiac targeting increased 3.2-fold; reduces systemic toxicity and improves bioavailability	[170]
	Pyrostat-HF	Oral selective GSDMD inhibitor; inhibits pyroptosis-related inflammatory cytokine release; improves myocardial remodeling and cardiac function	Phase IIb clinical / FDA Fast Track	Clear benefits for patients with heart failure; significantly improves LVEF and reduces heart failure biomarkers; Phase III pending	[158]
	OLT1177	Novel selective NLRP3 inflammasome inhibitor; blocks ASC/caspase-1 activation; inhibits pyroptosis-induced inflammatory storm	Early clinical	Higher oral bioavailability and lower cardiotoxicity compared with MCC950	[171]
	Piperlongumine (PL)	Inhibits NF-κB/NLRP3 signaling pathway activation; reduces secretion of pyroptosis-related inflammatory factors; alleviates myocardial inflammatory injury	Preclinical	Natural alkaloid; combines anti-inflammatory and anti-pyroptotic effects; suitable for viral myocarditis	[172]
Mitochondrial damage/mPTP	H-FAME	Cardiac-homing nanoformulation; inhibits UCP2, scavenges mitochondrial ROS; stabilizes mitochondrial membrane potential and mPTP opening	Preclinical	Simultaneously targets mitochondrial damage, cell necrosis, inflammation, and fibrosis; offers multidimensional cardioprotection	[160]
	Cyclophilin inhibitors	Non-immunosuppressive, highly selective inhibitors of cyclophilin D (CypD); blocks pathological mPTP opening	Preclinical	Higher potency than NIM811 and alisporivir; no immunosuppressive side effects	[173]
Multi-target broad-spectrum anti-necrotic	Compound 23a	Inhibits RIPK1, exerts potent antioxidant activity, and blocks lipid peroxidation; simultaneously antagonizes necroptosis and ferroptosis	Preclinical	Triple protective mechanism; significant therapeutic potential in myocardial ischemia–reperfusion injury	[174]
	ML355	Inhibits the ALOX12 pathway and modulates downstream TAK1/AMPK signaling; cross-species inhibition of myocardial programmed necrosis	Preclinical	Effective in mouse, large animal, and primate models; high clinical translation potential	[161]
	Spermidine	Activates the SIRT3 pathway, improves mitochondrial function; inhibits pyroptosis, oxidative stress, and drug-induced myocardial injury	Preclinical	Endogenous metabolite; high safety profile; applicable to the prevention/treatment of chemotherapy-induced cardiomyopathy	[175]
	saNppa-LNP	Lipid nanoparticle-mediated RNA delivery; long-term expression of ANP; inhibits myocardial necrosis and pathological remodeling	Preclinical	Long-lasting benefits after a single administration; minimally invasive with high targeting efficiency; novel gene intervention approach	[176]

Notes: ACSL4, acyl-CoA synthetase long-chain family member 4; ALOX12, arachidonate 12-lipoxygenase; ASC, apoptosis-associated speck-like protein containing a CARD; CypD, cyclophilin D; FTH1, ferritin heavy chain 1; GSDMD, gasdermin D; H-FAME, heart-homed fast-acting mitochondrial-function enhancer; HO-1, heme oxygenase-1; mPTP, mitochondrial permeability transition pore; NF-κB, nuclear factor kappa-light-chain-enhancer of activated B cells; NLRP3, NOD-like receptor family pyrin domain-containing 3; Nrf2, nuclear factor erythroid 2-related factor 2; RIPK3, receptor-interacting protein kinase 3; SIRT3, sirtuin 3; TAK1, transforming growth factor-β-activated kinase 1; UCP2, uncoupling protein 2; ZBP1, Z-DNA-binding protein 1; LVEF, left ventricular ejection fraction.

## 5. Challenges in Translating Research Findings to Clinical Practice and Future Research Directions

Despite significant advances in preclinical research, several challenges continue to hinder clinical translation. These include substantial redundancy in necrotic pathways, species-specific differences, safety concerns associated with long-term inhibition, and dynamic changes in specific necrosis subtypes across disease stages. Future research should focus on developing precision diagnostic tools, such as multi-omics technologies (proteomics, metabolomics, genomics, and transcriptomics), to identify specific biomarkers for distinct human necrosis subtypes. The development of multi-target drugs aimed at key crosstalk nodes (*e*.*g*., the inflammation-necrosis axis) is warranted. In addition, leveraging nanotechnology for precise drug delivery to the injured myocardium also represents a promising strategy.

## 6. Limitations

This article systematically reviews the signaling pathways and related therapeutic targets of autophagy, apoptosis, and various forms of PCD in cardiomyocytes. Nevertheless, certain limitations should be acknowledged. The review includes only English-language articles retrieved from the PubMed and Web of Science databases and excludes conference papers, unpublished literature, and studies published in non-English or less widely used languages, resulting in the omission of some recent and cutting-edge findings. Most existing studies are based on cellular and rodent models, and the potential species differences may limit the translational relevance to clinical practice. Additionally, the complex crosstalk among PCD signaling pathways has not yet been integrated into a unified regulatory network; therefore, this review provides only a summary of individual pathways. Moreover, most therapeutic targets remain at the preclinical stage and lack validation through large-scale clinical trials. Further investigation is required to determine the specificity, safety, and clinical applicability of targeted drugs.

## 7. Conclusions

Recent research has revealed that cardiomyocyte autophagy, apoptosis, and necrosis constitute a multilayered and dynamically integrated system that is deeply intertwined with metabolic states, the epigenetic landscape, inter-organelle communication, and other cell death pathways. The evolving understanding of the signaling pathways and regulatory mechanisms governing autophagy, apoptosis, and necrosis illuminates the path toward future targeted therapies and combinatorial strategies for more precise intervention within the cell death regulatory network. This progress holds the potential to more effectively protect cardiomyocytes and facilitate the repair of damaged myocardium in conditions such as cardiomyopathy, MI, and heart failure, ultimately improving patient outcomes.
